# Health Tract, No. 75

**Published:** 1862-05

**Authors:** 


### HEALTH TRACT, NO. 75.

### HOUSEKEEPING.

It may interest general readers to know the cost of housekeeping in New-York, among families in "moderate" or "easy" circumstances, exclusive of house-rent, dress, teachers' bills, and "summerings."

A family near Union Square, of nine persons, (five grown and four children under fifteen,) footed up the bills on the last day of 1861, as follows:

| | |
|---|---|
| Green-grocer, (vegetables, fruits, poultry, etc.,) | $256 |
| Grocer, (coffee, tea, sugar, etc.,) | 96 |
| Cook, | 96 |
| Housemaid, | 75 |
| Coal and wood, | 77 |
| Milk, | 76 |
| Meats, | 59 |
| Gas, | 36 |
| Sundries, of ice, repairs to range, etc., | 29 |
| | $800 |

This family lived in their own house; purchased all their supplies by the small quantity, excepting apples, flour, and fuel; as leaving less margin for waste, purloining and losses from decay of provisions; thus having every thing good and fresh of its kind. As to style and quality of the table: seven barrels of apples were provided in autumn; the flour always exceeded eight dollars a barrel; loaf-sugar was mainly used, except for pastries; butter averaged thirty-eight cents a pound; home-made bread; no liquors for drink; not a dollar for medicines; Java coffee roasted at home; tea, one dollar a pound; from three to four quarts of milk a day; nine gas-burners were lighted every night, sometimes ten or more, three of which always burned some all night; dessert of some kind every day, and more or less of company, eating and lodging, every week. Servants were required to be *in* bed before the clock struck ten; the area-gate was always kept locked; there were no perquisites of rags and soap-fat sold at two cents a pound, made of bacon, lard, and butter at forty cents; no cats or dogs were kept; neither ants, roaches, bed-bugs, nor musquitoes. Servants were selected who had been at their "last places" several years; who were middle-aged; looked tidy all the time, especially the cook; had no relations, and few indeed, if any visitors. Nothing was kept under lock and key.

Thus a family of eight or ten persons may live in comfort, cleanliness, and health in New-York, for eight hundred dollars a year, for fuel, lodging, washing, lights, food, and drink; guarding against wasteful, dishonest, and *liberal* servants, late hours, and costly, early, or out-of-season dishes.

It is painful to know how many families strive desperately and yet unavailingly, to "get ahead" in New-York City; the failure arising too often from their setting out with a style of living which was decided on in ignorance of what it ought to be; in consequence of forming an opinion from those who were living beyond their means, or who had resources greater than their own; then the strife to "keep up," involves those excessive efforts, those anxious toils and corroding cares which eat out all the joys of life, undermine the health, and make a premature grave, leaving children with no other heritage but the necessities of the same toils and cares, with the same false ambitions, and to make of life a failure also!

